# Determination of Intact Parabens in the Human Plasma of Cancer and Non-Cancer Patients Using a Validated Fabric Phase Sorptive Extraction Reversed-Phase Liquid Chromatography Method with UV Detection

**DOI:** 10.3390/molecules26061526

**Published:** 2021-03-11

**Authors:** Anthi Parla, Eirini Zormpa, Nikolaos Paloumpis, Abuzar Kabir, Kenneth G. Furton, Željka Roje, Victoria Samanidou, Ivana Vinković Vrček, Irene Panderi

**Affiliations:** 1 Laboratory of Pharmaceutical Analysis, Division of Pharmaceutical Chemistry, School of Pharmacy, National and Kapodistrian University of Athens, Panepistimiopolis, Zografou, 15771 Athens, Greece; anthiparla@hotmail.com (A.P.); eirini.zorba1@gmail.com (E.Z.); paloumpisnick@gmail.com (N.P.); 2 Department of Chemistry and Biochemistry, Florida International University, Miami, FL 33199, USA; akabir@fiu.edu (A.K.); furtonk@fiu.edu (K.G.F.); 3 Department for Plastic, Reconstructive and Aesthetic Surgery, University Hospital Dubrava, 10 000 Zagreb, Croatia; zroje@kbd.hr; 4 Laboratory of Analytical Chemistry, Department of Chemistry, Aristotle University of Thessaloniki, 54124 Thessaloniki, Greece; samanidu@chem.auth.gr; 5 Institute for Medical Research and Occupational Health, Ksaverska cesta 2, 10 000 Zagreb, Croatia; ivinkovic@imi.hr

**Keywords:** fabric phase sorptive extraction (FPSE), parabens, *p*-hydroxybenzoic acid esters, human plasma, bioanalysis

## Abstract

Parabens have been widely employed as preservatives since the 1920s for extending the shelf life of foodstuffs, medicines, and daily care products. Given the fact that there are some legitimate concerns related to their potential multiple endocrine-disrupting properties, the development of novel bioanalytical methods for their biomonitoring is crucial. In this study, a fabric phase sorptive extraction reversed-phase liquid chromatography method coupled with UV detection (FPSE-HPLC-UV) was developed and validated for the quantitation of seven parabens in human plasma samples. Chromatographic separation of the seven parabens and *p*-hydroxybenzoic acid was achieved on a semi-micro Spherisorb ODS1 analytical column under isocratic elution using a mobile phase containing 0.1% (*v*/*v*) formic acid and 66% 49 mM ammonium formate aqueous solution in acetonitrile at flow rate 0.25 mL min^−1^ with a 24-min run time for each sample. The method was linear at a concentration range of 20 to 500 ng mL^−1^ for the seven parabens under study in human plasma samples. The efficiency of the method was proven with the analysis of 20 human plasma samples collected from women subjected to breast cancer surgery and to reconstructive and aesthetic breast surgery. The highest quantitation rates in human plasma samples from cancerous cases were found for methylparaben and isobutylparaben with average plasma concentrations at 77 and 112.5 ng mL^−1^. The high concentration levels detected agree with previous findings for some of the parabens and emphasize the need for further epidemiological research on the possible health effects of the use of these compounds.

## 1. Introduction

Parabens are widely employed preservatives for extending the shelf life of foodstuffs, medicines, and daily care products [1]. These compounds are chemically stable without imparting any smell or taste and exhibit antimicrobial activity against a broad range of microorganisms. Such a combination of properties makes it difficult to find alternative preservatives to satisfactorily replace parabens. After ingestion, they are rapidly metabolized in the human intestine and liver into the relatively inactive metabolite, *p*-hydroxybenzoic acid, and its sulfuric acid and glucuronic conjugates, with less than 24 h biological half-life [2]. After dermal application, these compounds are partly metabolized to *p*-hydroxybenzoic acid by the skin enzymes [3], and the shorter alkyl chains derivatives cross the stratum corneum more easily than the longer chain derivatives [4]. Most studies showed that the estrogenic potential of the main metabolite of parabens, *p*-hydroxybenzoic acid, is weaker than that of the intact ingredients [5]. Although parabens are not mutagenic [6,7], there are concerns related to their potential multiple endocrine-disrupting action that are suspected to cause various health effects [8,9,10,11]. In the early 2000s, some studies indicated that the estrogenic activity of parabens increases with increasing the length of the linear alkyl chain group, with branching in the alkyl chain group or by the addition of a benzyl ring [12,13]. Further research has shown that despite the rapid metabolism rate, the concentration levels of the parent paraben esters in various human samples are not insignificant with the percentage of intact paraben esters excreted in urine to be dependent on the route of exposure [14,15]. The analysis of human breast tissue samples revealed that at least one intact paraben was present in 99% of the analyzed samples with a total median concentration at 85.5 ng/g [16]. In 2007, the Food and Agriculture Organization of the United Nations (FAO) and World Health Organization (WHO) Joint Expert Committee on Food Additives (JECFA) recommended the exclusion of propylparaben from use as a food preservative due to its adverse effects and allowed only the use of a group acceptable daily intake (ADI) of 0 to 10 mg/kg body weight (bw) for the sum of methylparaben and ethylparaben [17]. In the European Union, the allowed limit for propylparaben and butylparaben in cosmetics is 0.14% when used individually or together, while a safe concentration has been established for mixtures of parabens in cosmetics where the sum of the individual concentrations should not exceed 0.8% (as acid) [18]. Additionally, since 2014, isopropylparaben, isobutylparaben, benzylparaben, phenylparaben, and pentylparaben have been banned from use in cosmetics [19]. Recently, the Scientific Committee on Consumers Safety (SCCS) considered the concerns related to the potential endocrine-disrupting properties of propylparaben and concluded that the compound is safe when used as a preservative in cosmetic products up to a maximum concentration of 0.14% [20]. The concentrations of some parabens such as methylparaben, propylparaben, and the sum of parabens in umbilical cord plasma were associated to the levels of androgens in the same biofluid, and the widely used propylparaben was found to be negatively associated to the testosterone levels [21]. Recently, intact parabens have been detected in endometrial carcinoma tissues at a higher extent than in the normal endometrium, and propylparaben, isobutylparaben, and butylparaben were detected most frequently in all the tissue samples [22]. In another study contacted in Japan, the urine levels of parabens in pregnant women were measured in significantly high quantities indicating widespread exposure to parabens among these subjects [23]. Children and especially infants are vulnerable to the exposure of endocrine-disrupting chemicals (EDCs) in the environment due to their immature metabolic pathways [24]. Parabens are metabolized to *p*-hydroxybenzoic acid at different rates, and as a result, their exposure doses cannot be accurately assessed based only on their concentrations in human urine. Therefore, the development of novel bioanalytical methods for biomonitoring these compounds in human plasma has also been highlighted [25].

The literature survey revealed several chromatographic methods undertaken for the analysis of parabens in various biofluids [9,15,26,27,28,29]. These procedures were based on the use of liquid–liquid extraction (LLE) [30,31], solid phase extraction (SPE) [32], and several others sample preparation techniques [33,34,35,36,37]. Despite the extensive research in the quantitation of parabens in various biofluids, only a few published methods refer to the determination of the isomers of both propylparaben and butylparaben. These methods include a gas chromatographic-mass spectrometric (GC-MS) method for the quantitation of seven parabens in human breast milk [29,31] and liquid chromatographic methods coupled to diode array detection (HPLC-PDA) and the fabric phase sorptive extraction (FPSE) technique for the analysis of seven paraben residues in human whole blood, plasma, urine, and breast tissue samples [38,39,40].

In recent years, the development of novel sample preparation procedures following the philosophy of Green Analytical Chemistry (GAC) is a matter of growing interest among the analytical and bioanalytical scientists. To this purpose, we thought that it would be of interest to develop a method for the quantitation of seven parabens, namely methylparaben (MPB), ethylparaben (EPB), isopropylparaben (iPPB), propylparaben (PPB), butylparaben (BPB), isobutylparaben (iBPB), and benzylparaben (BzPB) in human plasma samples using a novel, eco-friendly, and efficient fabric phase sorptive extraction (FPSE) technique. FPSE simplifies the analytes extraction from complex matrices and reduces the solvent consumption. This technique utilizes a variety of fabric substrates chemically coated with sol–gel hybrid organic–inorganic sorbents, resulting in an efficient and eco-friendly sample pretreatment technique [41,42]. Since its discovery in 2014 by Kabir and Furton, FPSE has been applied to several analytes in variable samples including biological, environmental, and food samples, and by using various analytical techniques such as liquid chromatography-mass spectrometry (LC-MS), gas chromatography-mass spectrometry (GC-MS), and high-performance liquid chromatography with photodiode array detection (HPLC-PDA) [43,44,45]. In this work, the assay was based on the use of a small fraction (50 μL) of human plasma followed by an improved FPSE protocol using a sol–gel Carbowax^®^ 20M polar sorbent that exhibited improved sensitivity and reversed-phase HPLC-UV analysis on a semi-micro reversed phase analytical column. The method adequately separated the targeted analytes from their main metabolite, *p*-hydroxybenzoic acid [46], and it is in accordance with the green analytical chemistry philosophy. Finally, the proposed method was successfully applied to the analysis of human plasma samples taken from women subjected to malignant and benign plastic, reconstructive, and aesthetic breast surgery.

## 2. Results and Discussion

### 2.1. Method Development

#### 2.1.1. Chromatography Optimization

The chemical structures and the main physicochemical parameters that affect both chromatography and the FPSE procedure were calculated by ChemBioDraw ver. 13.0 (Perkin Elmer Informatics, Billerica, MA, USA) and are presented in Table 1. Several reversed-phase analytical columns have been tested for the chromatographic separation of the selected parabens, including Hypersil Gold^®^ C18 (150.0 mm × 2.1 mm, 5 μm), Spherisorb^®^ ODS1 (150.0 mm × 2.0 mm, 3 μm), and XTerraMS^®^ C18 (150.0 mm × 3.0 mm, 5 μm). Among these columns, both Hypersil Gold^®^ C18 and XTerraMS^®^ C18 did not allow the adequate separation of the isomers. On the other hand, Spherisorb^®^ ODS1 gives adequate separation for the selected seven parabens within a reasonable chromatographic runtime, and therefore, it was selected for this study. Consequently, chromatography was optimized for the adequate separation of the targeted analytes from matrix interferences within a chromatographic runtime of less than 24 min.

**Figure 1:**
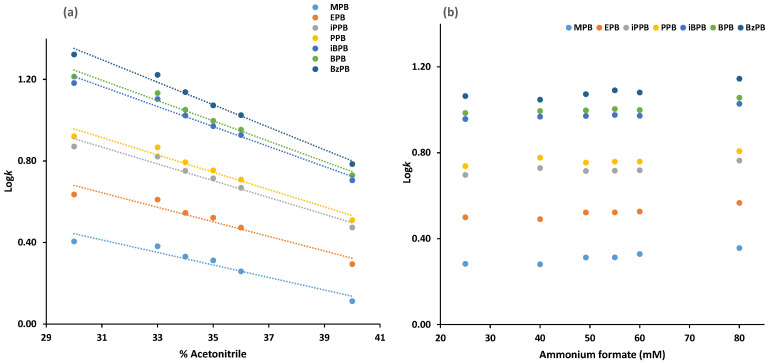
Plots of log*k* values of the targeted analytes versus (**a**) the percentage of acetonitrile and (**b**) ammonium formate concentration in the mobile phase.

Various combinations of ammonium formate aqueous solution mixed with acetonitrile or methanol with an altered content of each factor were examined to discover the optimal mobile phase. It was observed that acetonitrile, compared to methanol, decreases the retention of the targeted analytes and allows for their adequate separation from matrix interferences. The effect of the percentage of acetonitrile in the mobile phase, φ_ACN_, was evaluated in experiments where φ_ACN_ was varied from 30 to 40%, whereas ammonium formate concentration was kept constant at 31.5 mM in whole mobile phase and the percentage of formic acid was constant at 0.1% *v*/*v*. As it can be observed in Figure 1a, the increase in the percentage of acetonitrile yielded to a linear decrease in the retention factor (log*k* values) of the targeted analytes, as it was expected based on the retention mechanism of the reversed phase chromatography. Consequently, 34% acetonitrile was chosen as the optimum φ_ACN_ content in the mobile phase, as it allows adequate separation of all the analytes within less than 22 min.

With a constant acetonitrile content in the mobile phase at 34% and formic acid content at 0.1%, the concentration of ammonium formate was altered from 25 to 80 mM. Figure 1b indicates that an increase in the concentration of ammonium formate slightly increases the retention factors (log*k* values) of all parabens without affecting the separation of the isomers. In all tested mobile phases, the selected parabens are eluted in order of increased lipophilicity (Table 1). Thus, MPB, which is less lipophilic (LogP 1.46), is firstly eluted followed by EPB (LogP 1.83), iPPB (LogP 2.12), PPB (LogP 2.29), iBPB (LogP 2.69), BPB (LogP 2.71), and BzPB (LogP 3.20). It was also found that the column backpressure decreased, and analytes separation from matrix interferences was improved by increasing the ammonium formate concentration in the mobile phase. The best results achieved when a mobile phase consisting of 66% 49 mM ammonium formate aqueous solution in acetonitrile with 0.1% *v*/*v* formic acid was used for the separation of the targeted analytes. Column equilibration was achieved within 1 h, and the proposed isocratic LC method allows for adequate separation without the need for column re-equilibration.

**Figure 2:**
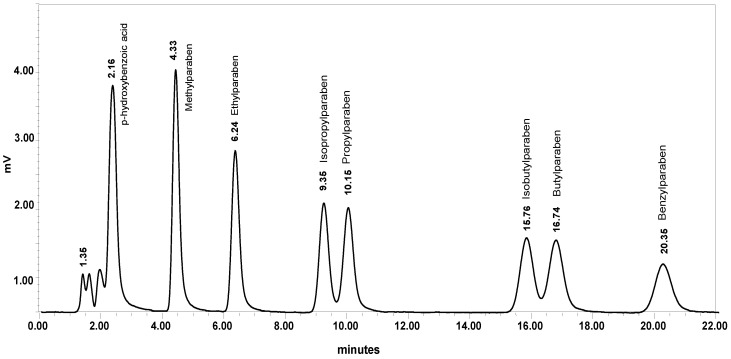
HPLC-UV chromatogram of a mixed working standard solution of the seven parabens at 100 ng mL^−1^ and *p*-hydroxybenzoic acid at 200 ng mL^−1^. Chromatographic conditions: Spherisorb^®^ ODS1 column; mobile phase: aqueous ammonium formate solution at 49 mM/acetonitrile (66:34, *v*/*v*) with 0.1% formic acid, column temperature 25 °C, flow rate 0.25 mL min^−1^ at 257 nm.

Figure 2 shows a chromatogram of a mixed standard solution of the seven parabens at 100 ng mL^−1^ and *p*-hydroxybenzoic acid at 200 ng mL^−1^ prepared in 60% 5 mM ammonium formate aqueous solution in acetonitrile (dilution solvent) and detected at 257 nm. Under the optimum chromatographic conditions, the seven parabens are well separated within less than 22 min, and their main metabolite, *p*-hydroxybenzoic acid, is eluted at 2.16 min and therefore, it does not interfere in their analysis.

#### 2.1.2. Optimization of the FPSE Procedure

Human plasma contains various ingredients, mainly proteins such as albumin, globulin, and fibrinogen, electrolytes, hormones, vitamins, lipids, and other substances. Due to this complex composition, an appropriate sample preparation procedure for removing matrix interferences is crucial prior to the chromatographic analysis. In addition, the optimization of a sample preparation procedure for the analysis of the selected parabens in human plasma can be challenging due to their varied polarity, logP values range from 2.08 for MPB to 3.28 for BzPB.

FPSE is a new, innovative, and promising technique for sample preparation. This technique uses a natural or synthetic fabric membrane as a substrate that is chemically coated in the form of a very thin but spongy coating of sol–gel organic–inorganic hybrid sorbent [47]. In this study, a sol–gel Carbowax 20M coated FPSE membrane was used for sample preparation of the human plasma samples [38,39]. This membrane consists of a biocompatible organic poly(ethylene glycol) polymer, an inorganic precursor, and a hydrophilic natural polymer cellulose fabric that synergistically complement each other to determine the overall selectivity of the extraction device. The FPSE membrane has been previously used for the analysis of parabens in various biofluids [35], including human plasma. However, in this study, the procedure was modified and optimized for maximum recovery of the analytes using a small volume of human plasma samples, intending to achieve increased sensitivity for the analysis of samples collected from women subjected to malignant and benign plastic, reconstructive, and aesthetic surgery of breasts as well as evaluate the presence of parabens. In this regard, several parameters were thoroughly investigated including the type and the volume of the extraction solvent, the extraction time, the desorption solvent, the desorption time, and the reconstitution solvent mixture. To achieve maximum extraction efficiency, the selected parameters (factors) were studied and evaluated at each stage of the technique, each time changing one factor and keeping the rest stable. In all the experiments, a small amount of human plasma samples (50 μL) was used spiked with the targeted analytes at a concentration of 250 ng mL^−1^, and the sol–gel Carbowax 20M FPSE membrane was cut into a size of 2.25 cm^2^. 

**Figure 3:**
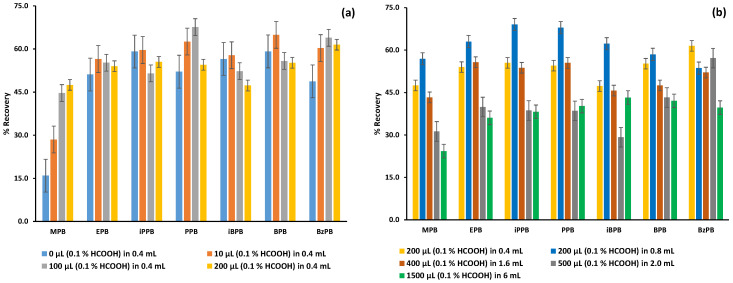
Influence of (**a**) the type of extraction solvent and (**b**) the extraction volume on the percentage recovery of the seven parabens; MPB for methylparaben, EPB for ethylparaben, iPPB for isopropylparaben, PPB for propylparaben, BPB for butylparaben, iBPB for isobutylparaben, and BzPB for benzylparaben.

The type and the volume of the extraction solvent are critical parameters in any FPSE procedure, and they were optimized to deliver the maximum percentage recovery for each analyte. As it is shown in Figure 3a, when an aliquot of 0.35 mL of water was used to extract 50 μL of a human plasma sample, the percentage recovery of the analytes ranged from 14.9% for MPB to 59.1% for iPPB. The seven parabens under study are in a neutral state under acidic conditions since their pKa values are around 8.9 (Table 1). Thus, to increase the interaction of the targeted analytes with the neutral extraction sorbent of the sol–gel Carbowax 20M-coated FPSE membrane, the extraction solvent should be acidified. Consequently, the aqueous extraction solvent was acidified by the addition of formic acid. It was found that the percentage recovery for MPB, which is the less lipophilic compound, increased radically and reached 47.5% after acidification of the extraction solution with 0.2 mL of a 0.1% formic acid aqueous solution.

To further improve the percentage recovery of the targeted analytes, various experiments have been performed where the total extraction volume varied from 0.4 to 6 mL, with varied content of 0.1% formic acid aqueous solution. As it is shown in Figure 3b, the maximum percentage recoveries for all the analytes were attained with an extraction volume of 0.8 mL acidified with 200 μL of 0.1% formic acid aqueous solution.

**Figure 4:**
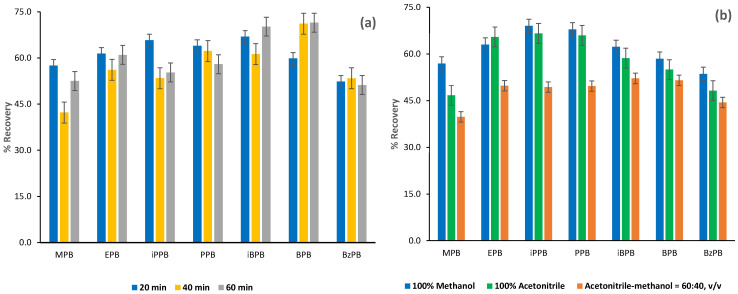
Influence of (**a**) the extraction time and (**b**) the type of the desorption solvent on the percentage recovery of the seven parabens; MPB for methylparaben, EPB for ethylparaben, iPPB for isopropylparaben, PPB for propylparaben, BPB for butylparaben, iBPB for isobutylparaben, and BzPB for benzylparaben.

During the extraction and desorption (back-extraction) procedure, the screw-capped glass vials were placed in a vertical rotating mixer with a reciprocal rotation speed set at 12 rpm and reciprocal rotation tilt angle range set at 20°. Three different time periods were tested for extraction, and as it is illustrated in Figure 4a, a 20-min extraction time gave the highest percentage recoveries for all the analytes. As it is illustrated in Figure 4b, the optimum percentage recovery for the targeted analytes is attained using 0.8 mL methanol as desorption (back-extraction) solvent. Three different time intervals have been tested for desorption—10, 20, and 30 min—and it was found that within 20 min, optimum percentage recovery is attained. After desorption, the methanolic eluent was evaporated to dryness under a gentle stream of nitrogen at 30 °C.

**Figure 5:**
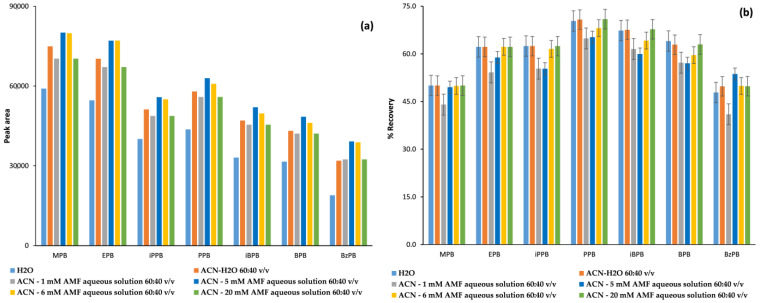
Influence of the reconstitution solvent on (**a**) the chromatographic response and (**b**) the percentage recovery of the seven parabens; MPB for methylparaben, EPB for ethylparaben, iPPB for isopropylparaben, PPB for propylparaben, BPB for butylparaben, iBPB for isobutylparaben and BzPB for benzylparaben.

In the early steps of method development, we have noticed that the reconstitution solvent affects the chromatographic response (peak area) of the analytes (Figure 5a). To this regard, various solvent mixtures have been tested for reconstitution of the samples after evaporation of the methanolic back-extraction solvent, including water, acetonitrile–water mixture 60:40, *v*/*v* and acetonitrile with various concentrations of ammonium formate aqueous solution 60:40, *v*/*v*. As it is clearly shown in Figure 5b, the optimum solvent mixture for reconstitution is acetonitrile/5mM ammonium formate aqueous solution 40/60, *v*/*v* as it gives the highest percentage recovery for the analytes with the lowest percentage recovery, namely MPB and BzPB.

The residues were reconstituted with 150 μL of the reconstitution solvent and then filtered through a syringe filter prior to HPLC-UV analysis. Various syringe filters such as nylon membrane, hydrophobic polytetrafluorethylene (PTFE), and hydrophilic PTFE have been tested for the final filtration step prior to the HPLC-UV analysis. A 13 mm hydrophilic PTFE membrane syringe filter with 0.22 μm pore size gave a percentage recovery greater than 98.0% for all the analytes, and therefore, it was selected as the optimum for the analysis of the seven parabens under study.

#### 2.1.3. Mechanism of Extraction in FPSE Membrane

Classical sample preparation techniques often utilize highly viscous polymeric sorbents such as poly(dimethylsiloxane). The sorption properties of these viscous sorbents toward the target analyte(s) are described by (a) solubility (S) and the partition coefficient (K). These two parameters define the relative concentration of the analyte at equilibrium between the polymeric sorbent and the sample matrix [48]. Due to the high viscosity of the polymer, the mass transfer of analyte between the sorbent and the sample matrix is relatively slow. However, the extraction of analytes in the FPSE membrane is governed by adsorption. Sol–gel sorbents are inherently porous, possessing sponge-like architecture containing many functional moieties that are highly affinitive toward the target analytes. When the FPSE membrane is inserted into the aqueous solution, the analytes approach toward the FPSE membrane via diffusion and interact with the sorbent via different intermolecular interactions such as London dispersion, dipole–dipole interaction, and hydrogen bonding. The planer geometry of the FPSE membrane, the sponge-like porous architecture of the sol–gel sorbent, and the built-in pores of the fabric substrate allow rapid permeation of the aqueous sample through the FPSE membrane. The continuous passage of the same sample through the FPSE membrane ensures fast extraction equilibrium and exhaustive/near-exhaustive extraction in a relatively short period.

**Figure 6:**
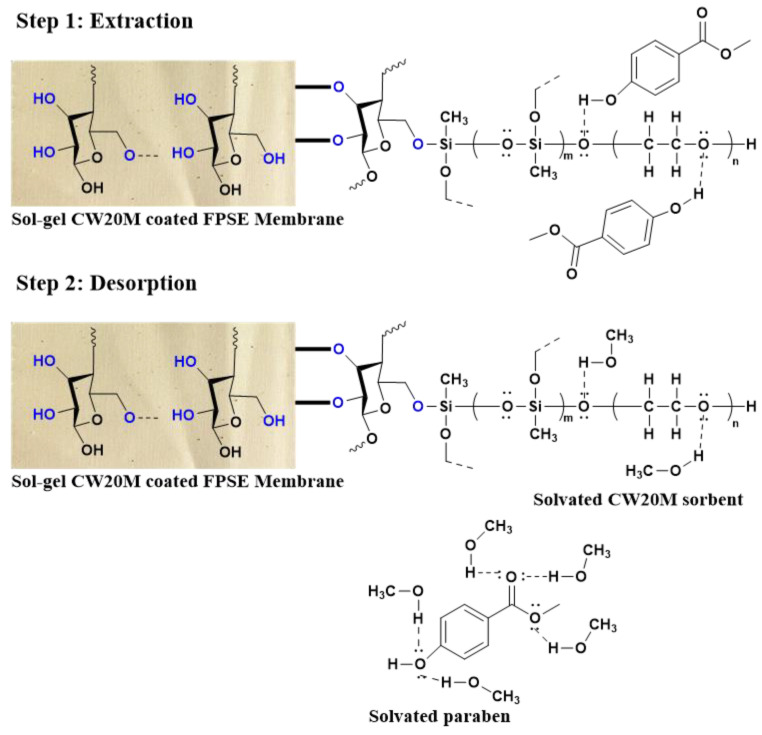
Schemes demonstrating extraction and desorption processes in fabric phase sorptive extraction.

At the end of extraction, the FPSE membrane is introduced into a small volume of organic sorbent such as methanol. The solvent solvates both the FPSE membrane as well as the analytes. As a result, the interaction between the sorbent and the analytes are shattered, and the analytes are solvated with methanol. Due to the high porosity and sponge-like internal structure of sol–gel sorbent, a small volume of methanol can quantitatively scavenge the analytes from the FPSE membrane very fast. Both the extraction and desorption processes are presented in Figure 6. 

#### 2.1.4. Green Attributes of Fabric Phase Sorptive Extraction

Fabric phase sorptive extraction was invented as a new generation green sample preparation technology. Galuszka et al. [49] compiled 12 principles of green analytical chemistry. Surprisingly, FPSE meets 10 out of 12 principles. One major green attribute of FPSE is the substantial reduction of steps involved in the overall sample preparation workflow. As such, FPSE has not only simplified the sample preparation process but also significantly reduced organic solvent consumption, eliminated sample pretreatment and post-treatment steps, and supported field deployability. FPSE is the only sample preparation technique that allows the deployment of custom membrane size based on the volume of sample to be analyzed. For biological samples, FPSE allows whole blood analysis without converting it into plasma or serum. Sample preparation without any sample pretreatment ensures minimal analyte loss during the sample preparation and improves the quality and confidence in analytical data.

### 2.2. Statistical Analysis of Method Validation Data

#### 2.2.1. Selectivity and Specificity

All the chromatograms obtained from the analysis of five blank plasma samples (negative control samples) contained no co-eluting peaks greater than 5% of the peak area of the targeted analytes at 20 ng mL^−1^ showing the selectivity of the chromatographic method. In addition, the carry-over test met the predefined requirements, as no interfering peaks with responses greater than 5% of the peak areas at 20 ng mL^−1^ of each analyte were detected in blank human plasma samples analyzed after a high concentration calibration standard spiked in human plasma.

**Figure 7:**
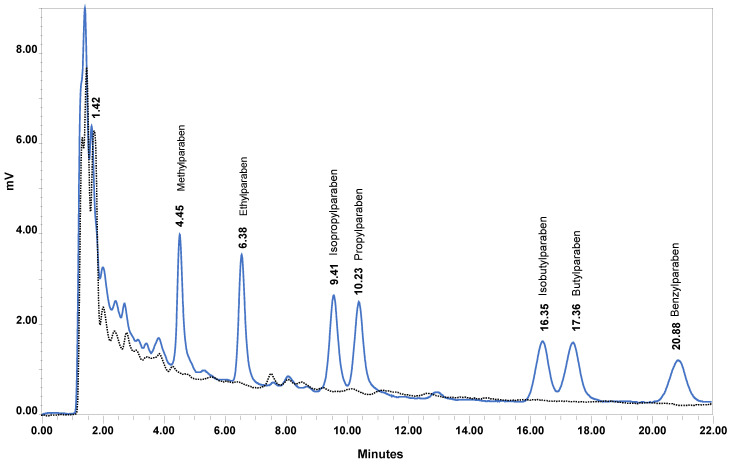
HPLC-UV chromatogram of a blank human plasma sample (black dotted line) overlaid with a chromatogram of a calibration plasma sample spiked with the targeted analytes at 200 ng mL^−1^ (blue line).

The developed FPSE-HPLC-UV method selectivity is further demonstrated in Figure 7, where a chromatogram of a blank plasma sample is superimposed with a chromatogram of a calibration plasma sample at 200 ng mL^−1^ for each analyte. MPB, EPB, iPPB, PPB, iBPB, BPB, and BzPB were eluted at 4.46, 6.39, 9.41, 10.33, 16.35, 17.36, and 20.88 min, respectively.

#### 2.2.2. Linearity, Precision, and Accuracy

A weighted linear regression analysis with a weighting factor of 1/y^2^ was adopted due to data heteroscedascity and because of the better results regarding other weighting factors (1/x, 1/x^2^) and unweighted linear regression, which was also tested. Data are presented in Table 2 and indicate that linear relationships were attained between the responses of the targeted analytes with regard to the corresponding concentrations. Back-interpolated concentrations in the calibration curves were less than 15.8% of the nominal concentration at lower limit of quantitation (LLOQ) levels, which is in agreement with international guidelines [50]. 

A series of dilute solutions of known concentration spiked in blank human plasma have been analyzed and limits of detection and quantitation (LOD and LOQ) for the targeted analytes were estimated based on signal-to-noise ratios of 3:1 and 10:1. The limits of detection, LOD, and the limits of quantitation, LOQ, were found to be at the level of 7 and 20 ng mL^−1^ for each analyte, respectively. 

One-way ANOVA was used for precision and accuracy evaluation, and the results are shown in Table 3. The precision and accuracy tests met the predefined requirements since the repeatability (intraday percentage CVs) ranged from 1.33 to 9.05% and the total accuracy ranged from 96.95 to 105.45%. 

#### 2.2.3. Recovery

The percentage relative recovery of the method for the seven parabens under study was also calculated as the percentage of the ratio of the slope of the regression equation of spiked human plasma samples to the slope of the regression equation of calibration samples spiked in water at equivalent concentrations (Table 2). All the samples have been analyzed in acetonitrile: 5 mM ammonium formate aqueous solution 40/60, *v*/*v* and the calibration spiked water samples have not been processed by the FPSE procedure (Table 2). Based on these data, percentage relative recoveries of 53.7, 57.7, 65.4, 60.6, 62.2, 62.1, and 50.5% were attained for MPB, EPB, iPPB, PPB, iBPB, BPB, and BzPB, respectively.

The percentage absolute recovery was determined by the percentage of the ratio of the peak area measured from human plasma samples spiked at 200 ng mL^−1^ for each analyte to the peak area of blank human plasma samples spiked after the FPSE procedure with analytes at equivalent concentrations. Based on this test, the percentage of absolute recoveries of 50.1, 60.2, 55.4, 65.8, 61.2, 56.9, and 53.3% were attained for MPB, EPB, iPPB, PPB, iBPB, BPB, and BzPB, respectively.

#### 2.2.4. Stability

Human plasma samples spiked with the analytes at 200 ng mL^−1^ and stored at ambient temperature for six hours remained constant. Percentage recoveries of the analytes ranged from 98.2 to 102.5%. Freeze and thaw stability of the targeted analytes was assessed by four consecutive freeze and thaw cycles applied to human plasma samples spiked with the analytes at 200 ng mL^−1^. The samples were frozen for 7 days at −18 °C and thawed at room temperature (one cycle); the procedure was repeated for three consecutive cycles. To calculate the stability, the data of the stored samples were compared to the data of freshly prepared human plasma samples spiked with the targeted analytes at 200 ng mL^−1^. Percentage recoveries of the analytes ranged from 97.5 to 102.6%, indicating that the analytes are stable after four complete freeze and thaw cycles.

### 2.3. Application to Real Human Plasma Samples

A total of 20 human plasma samples were analyzed, and the results are presented in Table 4 and Table 5. Half of the samples (*n* = 10 in the age range between 34 and 83) were collected from women subjected to malignant plastic surgery of breasts, and the rest (n = 10 ranging in age between 33 and 59) were collected from healthy women subjected to non-malignant benign reconstructive and aesthetic surgery of breasts.

The results indicate that MPB was quantified in 100% of the human plasma samples from cancerous cases with average plasma concentration at 77.0 ng mL^−1^. The highest quantitation rates in human plasma samples from cancerous cases were found for MPB (100%) and iBPB (40%) with average plasma concentrations at 77.0 and 112.5 ng mL^−1^, respectively. In healthy women, the highest quantitation rates were observed for both MPB (60%) and EPB (70%) with average plasma concentration at 60.3 and 50.0 ng mL^−1^, respectively. PPB was quantified only in cancerous cases at a rate of 20% with mean plasma concentration 20 ng mL^−1^, while iPPB was quantified both in healthy and cancerous cases with mean plasma concentration in all the samples at 44 ng mL^−1^. BPB was not quantified in any of the analyzed samples, while iBPB was quantified at a higher rate in cancerous cases (40%) than in healthy women (10%) with average plasma concentrations at 112.5 and 50 ng mL^−1^, respectively. The more lipophilic BzPB was quantified in both cancerous and healthy cases with mean plasma concentration in all the samples at 70 ng mL^−1^. As this was the pilot study that included a very small population size, statistical analysis was not performed, but the results are still indicative. As can be seen from Table 4, the mean age of woman in the group subjected to breast cancer surgery was higher by 10 years than for the group subjected to reconstructive and aesthetic surgery, but both groups were characterized by similar body mass index (BMI). In the human plasma samples obtained from the healthy women subjected to aesthetic reconstructive surgery, only MBP, EPB, and iPPB were detected, while iBPB and BzPBP were found in only single cases. On the other hand, PPB, iBPB, and BzPB were found in many more samples obtained from woman subjected to the breast cancer surgery. To undoubtedly determine the main factor for such differences, a much larger population group should be studied. Indeed, we will use the method presented here in future epidemiologic studies by evaluating the role of multiple factors on the accumulation of parabens in humans and the possible consequences of such accumulation. The high paraben concentrations detected in this study agree with the previous maximum concentration levels for MPB (142.9 ng mL^−1^), EPB (45.9 ng mL^−1^), and PPB (43.9 ng mL^−1^) reported by Sandanger et al. [25] in plasma samples of postmenopausal women. However, the latter study did not consider the analysis of the isomers, iPPB and iBPB. Furthermore, some studies have shown that conjugated parabens were stable in human serum over 30 days when stored at 37 °C [51], and thus, the contribution of conjugate hydrolysis is considered negligible to the values reported in the current study. Therefore, we agree with Sandager et al. [25] that the high concentration of intact parabens identified in our study is not caused by the hydrolysis of conjugated parabens. 

### 2.4. Comparison with Other Analytical Methods

The proposed FPSE-HPLC-UV method has been compared with other methods dedicated to the analysis of parabens in human plasma as reported in the literature. The results of this literature survey are presented in Table 6. Among the reported methods, only the current method and the FPSE-LC-DAD method [35] allows for the simultaneous quantitation of all seven parabens, including the isomers of PPB and BPB in human plasma samples. In this work, we have optimized both the FPSE protocol to reduce the analysis time and increase the percentage recovery and the chromatographic procedure to achieve adequate separation of all seven parabens within 22 min at a flow rate of 0.25 mL min^−1^. The method allows for the quantitation of the seven parabens at adequately low LOQ and LOD values with a linearity range that allows the quantitation of parabens in clinical samples using a small sample volume of 50 μL.

## 3. Materials and Methods

### 3.1. Chemical and Reagents

The following 4 parabens—MPB, EPB, PPB, and BPB—were purchased from Acros Organics part of Thermo Fisher Scientific (Geel, Belgium), the two isomers iPPB and iBPB were obtained from Alfa-Aesar part of Thermo Fisher Scientific (Ward Hill, MA, USA), BzPB was acquired from TCI America (Portland, OR, USA), and *p*-hydroxybenzoic acid was purchased from Sigma-Aldrich (Saint Louis, MO, USA). The HPLC grade solvents used in the current study were bought from E. Merck (Darmstadt, Germany). HPLC grade purified water was produced by using a Synergy^®^ UV water purification system (Merck Millipore, Burlington, MA, USA). FPSE membranes were synthesized by Kabir and Furton based on a procedure described elsewhere [38,52]. Hydrophobic polytetrafluorethylene membrane syringe filters (PTFE phobic, 13mm, pore size 0.22 μm) were obtained from RephiLe Bioscience Ltd Europe, Novalab representative (Athens, Greece).

### 3.2. Human Plasma Samples

Human plasma samples were obtained from Dubrava’s University Hospital in Zagreb, Croatia. Particularly, human plasma samples were collected from 10 women subjected to malignant and 10 woman subjected benign plastic, reconstructive, and aesthetic surgery of breasts to evaluate the presence of parabens. The group subjected breast cancer surgery was characterized by the mean age of 56.9 ± 15.5 (in the range of 34 to 83 years old) and the mean body mass index (BMI) of 25.7 ± 3.9. The group subjected to plastic, reconstructive, and aesthetic surgery of breasts has the mean age of 47.4 ± 7.5 (in the range of 33 to 59 years old) and mean BMI of 25.5 ± 4.7. From each study participant, whole blood was collected from decubital vein in BD vacutainer with K_2_EDTA. Cells were removed by centrifugation for 15 min at 2000 × *g*, and the resulting plasma samples were immediately dispensed into 0.5 mL aliquots, stored, and transported at −20 °C, to avoid freeze–thaw cycles. Samples that were hemolyzed, icteric, or lipemic were excluded from the study. The survey was accepted and approved by the Ethical Committee of Dubrava’s University Hospital and University of Zagreb (380-59-10106-14-4290/82), School of Medicine, Croatia. Informed consents were acquired from all participants before any other action. Blank human plasma samples were taken from National Red Cross General Hospital in Athens, Greece. 

### 3.3. Instrumentation

The analytical instrument used was a Waters^®^ HPLC system (Waters, Milford, MA, USA), consisting of a Waters^®^ 717 plus auto sampler, a temperature oven, a Waters^®^ 1515 isocratic pump, and a Waters^®^ 486 UV detector operated at λ 257 nm, whereas Empower™ Chromatography Data System (Waters Chromatography Europe BV, Etten-Leur, The Netherlands) was used for data acquisition and analysis. The selected parabens were separated on a Spherisorb ODS1 C18 analytical column (150.0 × 2.0 mm i.d., particle size 3 μm) (Waters, Ireland). A mobile phase of 66% 49 mM ammonium formate water solution in acetonitrile containing 0.1% (*v*/*v*) formic acid was used at a flow rate of 0.25 mL min^−1^. The mobile phase was always degassed under vacuum while filtering through a 0.45 μm nylon membrane filter prior to use. Chromatographic experiments were performed at ambient temperature with a chromatographic run time of less than 24 min; each sample was injected into a 10 μL loop. A vertical rotating mixer model RS 2O-VS, Heto Lab Equipment, Heto-Holten A/S (Allerød, Denmark) was used for gentle mixing of the samples during FPSE procedure with reciprocal rotation speed set at 10 rpm and reciprocal rotation tilt angle range set at 20° and a Techne Sample concentrator Dri-block DB3 model FDB03OD (Techne Duxford, Cambridge, UK) was used for solvent evaporation.

### 3.4. Stock and Working Standard Solutions

Stock standard solutions of the analytes at 500 μg mL^−1^ were prepared in methanol. Mixed working standard solutions of the targeted analytes were prepared at the concentration range 25 to 2500 ng mL^−1^ by further dilutions of the stock solutions in water. The stock standard solutions were found stable when stored at −20 °C for 4 months, while the working standard solutions stored at 4 °C were prepared freshly every month.

### 3.5. Calibration Standards and Quality Control Samples

Calibration standards were prepared in human plasma at 20, 40, 60, 100, 200, 400, and 500 ng mL^−1^ for each analyte. Quality control (QC) was prepared at 20, 100, and 500 ng mL^−1^ in human plasma using separate stock solutions.

### 3.6. Sample Preparation Procedure

After thawing at room temperature, the samples are vortex mixed to ensure homogeneity prior to the sample preparation procedure, which is performed by an optimized FPSE procedure. The sol–gel Carbowax 20M membrane is cut at a size of 2.25 cm^2^ (1.5 × 1.5 cm) and immersed in 2 mL acetonitrile–methanol mixture 50:50, *v*/*v* for 5 min and then in 2 mL of deionized water for an additional 5 min. Consequently, the FPSE membrane is transferred into a 5 mL screw-capped glass vial, followed by the addition of 50 μL aliquot of each human plasma sample, 550 μL of deionized water, and 200 μL of 0.1% aqueous formic acid. Then, the vial is placed in a vertical rotating mixer with reciprocal rotation speed set at 12 rpm and a reciprocal rotation tilt angle range set at 20° for 20 min. Accordingly, the FPSE membrane is transferred into a clean 5 mL screw-capped glass vial containing 800 μL methanol and then placed in the vertical rotating mixer for 20 min for the elution of the analytes. The methanolic eluent is transferred into an Eppendorf tube, and the content is evaporated within 10 min under a gentle stream of nitrogen at 30 °C. The residue is reconstituted with 150 μL acetonitrile/5mM ammonium formate 40/60, *v*/*v* and then filtered using a 13 mm PTFE hydrophilic membrane syringe filter with a pore size of 0.22 μm prior to the chromatographic analysis.

### 3.7. Method Validation

The proposed FPSE-HPLC-UV method was validated for selectivity, specificity, linearity, limit of detection (LOD), lower limit of quantitation (LLOQ), repeatability (intraday precision), interday precision, overall accuracy, absolute recovery, matrix effect, and stability [50]. To evaluate the selectivity over any matrix interference, five blank human plasma samples (negative controls) obtained from different batches have been analyzed following the optimum conditions of the method. Matrix interference was investigated at the retention time window of each paraben set at 5% of the corresponding retention time. The absence of matrix interference is confirmed when the area of any interference is less than 5% of the area determined at the LLOQ level for each analyte. To evaluate the linearity, weighted least-squares linear regressions were used to construct the calibration graphs after the analysis human plasma calibration standards spiked at seven different concentration levels. The quantitation was performed measuring the peak area of each targeted analyte. The analyte response at the LLOQ level should be at least 5 times the signal of a blank sample. Precision and accuracy were established by analyzing four replicates of QC samples at three concentration levels and on three different days. Repeatability (intraday precision) and interday precision were evaluated by calculating the percentage coefficient of variations (% CVs), which based on the acceptance criteria should be less than 15%, and at the LOQ levels should be less than 20%. Overall accuracy was assessed by measuring the percentage relative recovery, and according to predefined criteria, the mean concentration should be within 15% of the nominal values for the QC samples, except for the LLOQ, which should be within 20% of the nominal value.

## 4. Conclusions

In the current study, the advantages of FPSE on the sample preparation of analytes with diverse lipophilicity and on the enhancement of the sensitivity of the HPLC-UV technique in bioanalysis were demonstrated through the development of an FPSE-HPLC-UV method for the quantitation of seven parabens in human plasma samples. The new and optimized FPSE protocol requires only 50 μL of biological sample for the extraction, allows for the quantitation of the seven parabens at an LOQ of 20 ng mL^−1^ and combined with a semi-micro reversed phase analytical column is in accordance with the philosophy of the Green Analytical Chemistry. The efficiency of the method has been proven with the analysis of the seven parabens in 20 real plasma samples obtained from healthy and cancerous cases. The results for the shorter alkyl chain parabens have shown that MPB was quantified in highest rates in both cancerous and non-cancerous cases, while EPB was present at the highest rates in healthy women, and PPB was present only in cancerous cases. The results for the longer alkyl chain parabens have shown that BPB was not quantified in any of the analyzed samples, while BzPB was quantified at 15% of all the samples. As for the isomers, iPPB was quantified in both cancerous and non-cancerous cases, and iBPB was quantified at the highest quantitation rates in cancerous cases. The high concentration levels detected agree with previous findings for some of the parabens and emphasize the need for further epidemiological research on the possible health effects of the use of these compounds.

## Figures and Tables

**Table 1:** Chemical structures and physicochemical parameters of the seven parabens under study.

Chemical Structures/Chemical Names	Physicochemical Parameters ^1^
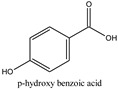	Log P: 1.2 CLogP: 1.5572 pKa: 4.109, 9.685
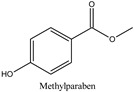	Log P: 1.46 ± 0.47 CLogP: 1.9846 pKa: 9.007
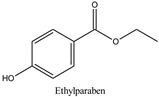	Log P: 1.83 ± 0.49 CLogP: 2.5136 pKa: 8.981
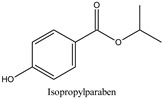	Log P: 2.12 ± 0.47 CLogP: 2.8226 pKa: 8.955
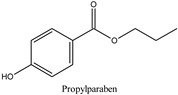	Log P: 2.29 ± 0.47 CLogP: 3.0426 pKa: 8.970
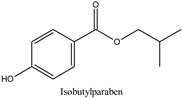	Log P: 2.69 ± 0.47 CLogP: 3.4416 pKa: 8.959
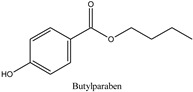	Log P: 2.71 ± 0.47 CLogP: 3.5716 pKa: 8.965
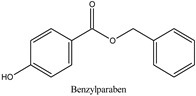	Log P: 3.20 ± 0.47 CLogP: 3.8126 pKa: 8.931

^1^ Physicochemical parameters were calculated by ChemBioDraw ver. 13.0.

**Table 2:** Linearity data for the quantitation of the seven parabens in human plasma samples as assessed by the fabric phase sorptive extraction reversed-phase liquid chromatography method coupled with UV detection (FPSE-HPLC-UV) method.

Compound ^1^	Matrix	Regression Equations ^2^	r ^3^	Standard Deviation		S_r_^4^
				Slope	Intercept	
MPB	*Water*	S_MPB_ = 113.9 × C_MPB_ −795	0.998	2.6	92	0.05
	*Human plasma*	S_MPB_ = 61.2 × C_MPB_ −598	0.996	2.4	78	0.08
EPB	*Water*	S_EPB_ = 122.1 × C_EPB_ −805	0.9994	1.8	65	0.04
	*Human plasma*	S_EPB_ = 70.4 × C_EPB_ −477	0.998	2.2	70	0.06
iPPB	*Water*	S_iPPB_ = 96.4 × C_iPPB_ −774	0.991	4.1	145	0.08
	*Human plasma*	S_iPPB_ = 63.1 × C_iPPB_ −479	0.993	3.4	121	0.11
PPB	*Water*	S_PPB_ = 97.5 × C_PPB_ −669	0.995	4.4	164	0.08
	*Human plasma*	S_PPB_ = 59.1 × C_PPB_ −429	0.993	3.1	116	0.10
iBPB	*Water*	S_iBPB_ = 98.1 × C_iBPB_ −789	0.998	2.8	99	0.08
	*Human plasma*	S_iBPB_ = 61.0 × C_iBPB_ −557	0.995	2.6	86	0.09
BPB	*Water*	S_BPB_ = 97.5 × C_BPB_ – 907	0.9991	2.2	72	0.05
	*Human plasma*	S_BPB_ = 60.7 × C_BPB_ – 566	0.995	2.5	82	0.09
BzPB	*Water*	S_BzPB_ = 92.5 × C_BzPB_ −682	0.998	2.4	87	0.05
	*Human plasma*	S_BzPB_ = 46.7 × C_BzPB_ −318	0.998	1.2	45	0.05

^1^ MPB for methylparaben, EPB for ethylparaben, iPPB for isopropylparaben, PPB for propylparaben, BPB for butylparaben, iBPB for isobutylparaben, and BzPB for benzylparaben; ^2^ Peak area signal of each paraben, S, versus the corresponding concentration, C, and over the concentration range 20 to 500 ng mL^−1^; ^3^ Correlation coefficient; ^4^ Standard error of the estimate.

**Table 3:** Accuracy and precision of the seven parabens in human plasma samples at three concentration levels as assessed by the FPSE-HPLC-UV method (*n* = three runs; four replicates per run).

Compound ^1^	Concentration (ng mL^−1^)		
**Added Concentration**	**20**	**100**	**500**
**MPB**			
Overall mean	20.88 ± 0.45	98.4 ± 3.5	494.2 ± 9.6
Intraday CV(%) ^2^	1.93	3.60	1.95
Total precision CV (%) ^2^	2.29	3.54	1.93
Total accuracy Er% ^3^	104.4	98.4	98.8
**EPB**			
Overall mean	20.94 ± 0.82	100.5 ± 4.9	504.6 ± 9.1
Intraday CV(%) ^2^	4.30	4.33	1.98
Total precision CV (%) ^2^	3.76	5.15	1.73
Total accuracy Er% ^3^	104.3	100.5	100.9
**iPPB**			
Overall mean	20.84 ± 0.46	100.2 ± 2.8	499.2 ± 8.9
Intraday CV(%) ^2^	6.54	3.05	1.60
Total precision CV (%) ^2^	5.90	2.66	1.86
Total accuracy Er%^3^	104.2	100.2	98.8
**PPB**			
Overall mean	20.4 ± 1.7	101.5 ± 3.2	500.2 ± 7.9
Intraday CV(%) ^2^	8.61	3.34	1.56
Total precision CV (%) ^2^	8.29	2.97	1.61
Total accuracy Er%^3^	102.0	101.5	100.1
**iBPB**			
Overall mean	21.1 ± 1.8	98.2 ± 3.8	501.5 ± 9.6
Intraday CV(%) ^2^	9.05	4.17	2.83
Total precision CV (%) ^2^	8.73	3.76	2.58
Total accuracy Er% ^3^	105.1	98.2	100.3
**BBP**			
Overall mean	19.39 ± 0.55	101.2 ± 1.6	498.8 ± 6.3
Intraday CV(%) ^2^	2.71	1.67	1.27
Total precision CV (%) ^2^	2.87	1.52	1.26
Total accuracy Er% ^3^	96.9	101.2	99.8
**BzBP**			
Overall mean	21.09 ± 0.55	103.9 ± 1.6	502 ± 11
Intraday CV(%) ^2^	2.82	1.56	2.50
Total precision CV (%) ^2^	2.53	1.50	2.33
Total accuracy Er% ^3^	105.4	103.9	100.4

^1^ MPB for methylparaben, EPB for ethylparaben, iPPB for isopropylparaben, PPB for propylparaben, BPB for butylparaben, iBPB for isobutylparaben, and BzPB for benzylparaben; ^2^ Coefficient of variation; intra- and inter-assay CVs were calculated by ANOVA; ^3^ Relative recovery percentage.

**Table 4:** Results on the concentration levels (in ng mL^−1^) of the seven parabens in human plasma samples collected from 20 women in Croatia.

Woman No.	Surgery ^1^	Age	BMI ^2^	Compound ^3,4^						
				MPB	EPB	iPPB	PPB	iBPB	BPB	BzPB
1	BC	43	25.0	110	40	50	<LOD	<LOD	<LOD	<LOD
2	BC	59	34.3	40	<LOD	30	<LOD	40	<LOD	<LOD
3	BC	34	28	150	<LOD	detected	<LOD	80	<LOD	70
4	BC	56	25.5	60	<LOD	40	<LOD	<LOD	<LOD	<LOD
5	BC	76	26	170	<LOD	detected	<LOD	<LOD	<LOD	<LOD
6	BC	45	20.9	30	detected	<LOD	detected	<LOD	<LOD	<LOD
7	BC	45	20.4	20	30	<LOD	30	30	<LOD	<LOD
8	BC	83	27.5	140	<LOD	<LOD	10	300	<LOD	90
9	BC	65	23	20	<LOD	<LOD	detected	<LOD	<LOD	<LOD
10	BC	63	26.2	30	detected	<LOD	<LOD	<LOD	<LOD	<LOD
11	AE	46	20.3	150	80	30	<LOD	50	<LOD	<LOD
12	AE	51	28.3	80	detected	70	<LOD	<LOD	<LOD	50
13	AE	48	18.4	detected	60	<LOD	<LOD	<LOD	<LOD	<LOD
14	AE	33	25.1	detected	20	<LOD	<LOD	<LOD	<LOD	<LOD
15	AE	47	27.6	detected	70	<LOD	<LOD	<LOD	<LOD	<LOD
16	AE	38	25.9	30	detected	<LOD	<LOD	<LOD	<LOD	<LOD
17	AE	59	24.9	60	40	<LOD	<LOD	<LOD	<LOD	<LOD
18	AE	55	35.4	detected	30	<LOD	<LOD	<LOD	<LOD	<LOD
19	AE	48	26.6	20	detected	<LOD	<LOD	<LOD	<LOD	<LOD
20	AE	49	22.6	22	50	<LOD	<LOD	<LOD	<LOD	<LOD

^1^ Type of surgery: BC for breast cancer; AE for aesthetic reconstructive surgery; ^2^ BMI for body mass index; ^3^ MPB for methylparaben, EPB for ethylparaben, iPPB for isopropylparaben, PPB for propylparaben, BPB for butylparaben, iBPB for isobutylparaben, and BzPB for benzylparaben; ^4^ Samples where parabens were detected but not quantified are denoted as “detected”, while the samples where parabens levels were below the limit of detection are denoted as “<LOD”.

**Table 5:** Results of the FPSE-HPLC-UV analysis of the seven parabens in human plasma samples collected from 20 women in Croatia.

Compound ^1^	MPB	EPB	iPPB	PPB	iBPB	BPB	BzPB
Percentage of the human plasma samples in which quantified and detected	100	60	30	30	25	Not detected	15
Percentage of the human plasma samples in which quantified	80	45	25	10	25	Not detected	15
Percentage of the human plasma samples from healthy women in which quantified	60	70	20	0	10	Not detected	10
Percentage of the human plasma samples from cancerous cases in which quantified	100	20	30	20	40	Not detected	20
Mean plasma concentration in heathy women (ng mL^−1^)	60.3	50.0	50.0	-	50.0	Not detected	50.0
Mean plasma concentration in cancerous cases (ng mL^−1^)	77.0	35.0	40.0	20.0	112.5	Not detected	80.0
Mean plasma concentration in all the samples (ng mL^−1^)	70.8	46.7	44.0	20.0	100.0	Not detected	70.0

^1^ MPB for methylparaben, EPB for ethylparaben, iPPB for isopropylparaben, PPB for propylparaben, BPB for butylparaben, iBPB for isobutylparaben, and BzPB for benzylparaben.

**Table 6:** Comparison of the proposed method with methods published in the literature for the quantitation of parabens in human plasma samples.

Matrix	Analytes	Analytical Method; Column; Flow Rate	Run Time	Sample Preparation/Extraction Time	Sample Volume	%Recovery	Repeatability (%CV)	Linearity Range	LOQ; LOD	Application to Real Samples	Reference
Human plasma	MPB, EPB, iPPB, PPB, iBPB, BPB, BzPB	RPHPLC-UV; Spherisorb ODS1 (150 × 2.0 mm, 3 μm); 0.25 mL/min isocratic elution	22 min	FPSE/ 40 min	50 μL	50.1–65.8%	1.3 to 9.0% (7 parabens)	20–500 ng/mL	LOQ: 20 ng/mL, LOD: 7 ng/mL	20 samples from healthy and cancerous patients (women)	Current method
Human plasma, whole blood, human urine	MPB, EPB, iPPB, PPB, iBPB, BPB, BzPB	RPHPLC-DAD; Spherisorb C18 (150 × 4.6 mm, 5 μm); 1.0 mL/min Isocratic elution	25 min	FPSE/ 60 min	450 μL	−	1.2 to 10.1% (7 parabens)	0.1–10 μg/mL	LOQ: 100 ng/mL, LOD: 30 ng/mL	6 samples	[38]
Human plasma	MPB, EPB, PPB, BPB, BzPB Bisphenols Estrogens	LC-MS/MS; Kinetex C18 (150 × 3.0 mm, 1.7 μm) 0.4 mL/min Gradient elution	11 min	LLE, derivatization with dansyl chloride	500 μL	103.6–112.7%	1.3 to 6% (5 parabens)	MPB: 0.25–32 ng/mL EPB, PPB, BPB, BzPB: 0.094–12 ng/mL	LOQ: 0.134 to 0.202 ng/mL	58 samples from men of reproductive age; 27 maternal and cord plasma samples	[21,31]
Human urine, serum, seminal plasma	MPB, EPB, PPB, BPB, BzPB	LC–MS/MS; Synergi™ Fusion-RP 80 Å (75 × 2.0 mm; 4 μm); 0.3 mL/min Gradient elution	17 min	Automated SPE/time not specified	500 μL	98.3–101.5%	2.8 to 29.2% (5 parabens)	0.5–500 ng/mL	LODs < 0.41 ng/mL	60 samples from young Danish men	[15]
Human plasma	MPB, EPB, PPB, BPB, BzPB	LC-TOF/MS; Waters® Acquity BEH Phenyl (100 mm × 2.1 mm, 1.7 μm) 0.45 mL/min Gradient elution	10 min	SPE on OASIS HLB cartridges	500 μL	-	< 12% (5 parabens)	50 to 600 pg injected on column	LODs: MPB 7 ng/mL EPB 3 ng/mL PPB 2 ng/mL	332 samples from postmenopausal women	[28]

## Data Availability

The data presented in this study are available on request from the corresponding author.

## Sample Availability

Samples of the compounds are not available from the authors.
